# Diversity of sialic acids and sialoglycoproteins in gametes and at fertilization

**DOI:** 10.3389/fcell.2022.982931

**Published:** 2022-10-20

**Authors:** Ingrid Fliniaux, Guillaume Marchand, Caroline Molinaro, Mathieu Decloquement, Alain Martoriati, Matthieu Marin, Jean-François Bodart, Anne Harduin-Lepers, Katia Cailliau

**Affiliations:** Univ. Lille, CNRS, UMR 8576-UGSF-Unité de Glycobiologie Structurale et Fonctionnelle, Lille, France

**Keywords:** sialic acid, glycoprotein, oocyte, sperm, egg, fertilization

## Abstract

Sialic acids are a family of 9-carbon monosaccharides with particular physicochemical properties. They modulate the biological functions of the molecules that carry them and are involved in several steps of the reproductive process. Sialoglycoproteins participate in the balance between species recognition and specificity, and the mechanisms of these aspects remain an issue in gametes formation and binding in metazoan reproduction. Sialoglycoproteins form a specific coat at the gametes surface and specific polysialylated chains are present on marine species oocytes. Spermatozoa are submitted to critical sialic acid changes in the female reproductive tract facilitating their migration, their survival through the modulation of the female innate immune response, and the final oocyte-binding event. To decipher the role of sialic acids in gametes and at fertilization, the dynamical changes of enzymes involved in their synthesis and removal have to be further considered.

## 1 Introduction

In vertebrate and invertebrate metazoan sexual reproduction, gametes acquire competence for fertilization during oogenesis and spermatogenesis. Before fertilization, both male and female gametes have to go through a long journey in aquatic environments or into the urogenital tracts, and have therefore developed specificity in the envelopes surrounding their plasma membrane. Gametes are surrounded by a specific extracellular matrix that exerts several functions. One function is to protect them from external (freshwater or seawater) or internal (female genital tract: vagina-cervix, uterus, oviduct) environments. This cellular matrix is enriched in negatively charged glycoconjugates, ending with sialic acids (Sias) (Schauer, 2004; Schauer, 2009). Sias predispose gametes to hydration protection, necessary for gametes interaction and successful fertilization (Hirohashi et al., 2008; Li et al., 2011). In the oocyte jelly coat, Sias also play a role in water retention and are a reservoir for several ions including calcium (Berner and Ingermann, 1990). By virtue of both bulk and negative charge, large acidic polymers of polysialic acids (polySias) modulate negatively cell to cell and cell to matrix interactions (Varki, 1992) and protect gametes before fertilization. Sialylated glycan chains on proteins occupy more space compared to a naked globular protein (Cyster et al., 1991; Li et al., 2011; Bhide and Colley, 2017), and form a cap impairing interaction with plasma membrane conjugates that limit species unspecific fertilization. However, sialoglycoproteins are also involved in specific species recognition between the spermatozoa and the oocyte. Moreover, a balance is maintained between species specificity and immune protection through the occurrence of a spermatozoa dynamical change in the Sias coat in vertebrates and invertebrates. For those reasons, fertilization is more than a complex series of Sias modifications and interactions compared to the general recognition mechanism found in pathogens using Sias residues on their carbohydrates (as for viruses, Glasner et al., 2012; Rosenstock and Kaufmann, 2021).

The present review highlights the diversity and properties of Sias present on glycoproteins to ensure their multiple roles in gametes and at fertilization. To get further insight into the specificity of polySias in both oocytes and spermatozoa, the structure, biosynthesis of glycoproteins, and the phylogenic filiation of sialyltransferases are considered. The presence and the species-specific composition in the surrounding membranes of the oocytes with polySias chains diversity in aquatic species and the specificity of sialylated ZP in the mammalian *zona pellucida* (ZP) are then addressed. A third part is dedicated to the coating and uncoating fine-tuned regulation of Sias in sperm. Remodeling the surrounding coat of sialoglycoproteins finalizes the spermatozoa fertility properties. In addition, specific interactions between sperm Sias and Siglec receptors (sialic acid-binding immunoglobulin-like lectins) (Tecle et al., 2019) occur in the female genital tract of mammals counteracting the female immune response. In a last part, the final unmasking of Sias from the oocyte coat by the spermatozoa acrosomal reaction to gain accessibility when the gametes encounter is discussed at fertilization.

## 2 Sias structure and biosynthesis

### 2.1 Sias structural diversity

Gametes surfaces, like other cell surfaces, are covered by a thick dense coat known as the glycocalyx, formed by a complex network of glycoconjugates including glycoproteins and glycolipids representing up to 30 μm for echinoderm oocytes (Cohen and Varki, 2010; Schjoldager et al., 2020). The terminal position of oligosaccharide chains (i.e. glycans) is occupied by a variety of negatively charged acidic monosaccharides, the Sias, all derived from Neu5Ac (*N*-Acetylneuraminic acid) and Kdn (2-keto-3-deoxynononic acid). Interestingly, these sialylated glycoconjugates are characterized by a high structural and functional diversity, which can be analyzed at different levels (Cohen and Varki, 2010).

**FIGURE 1:**
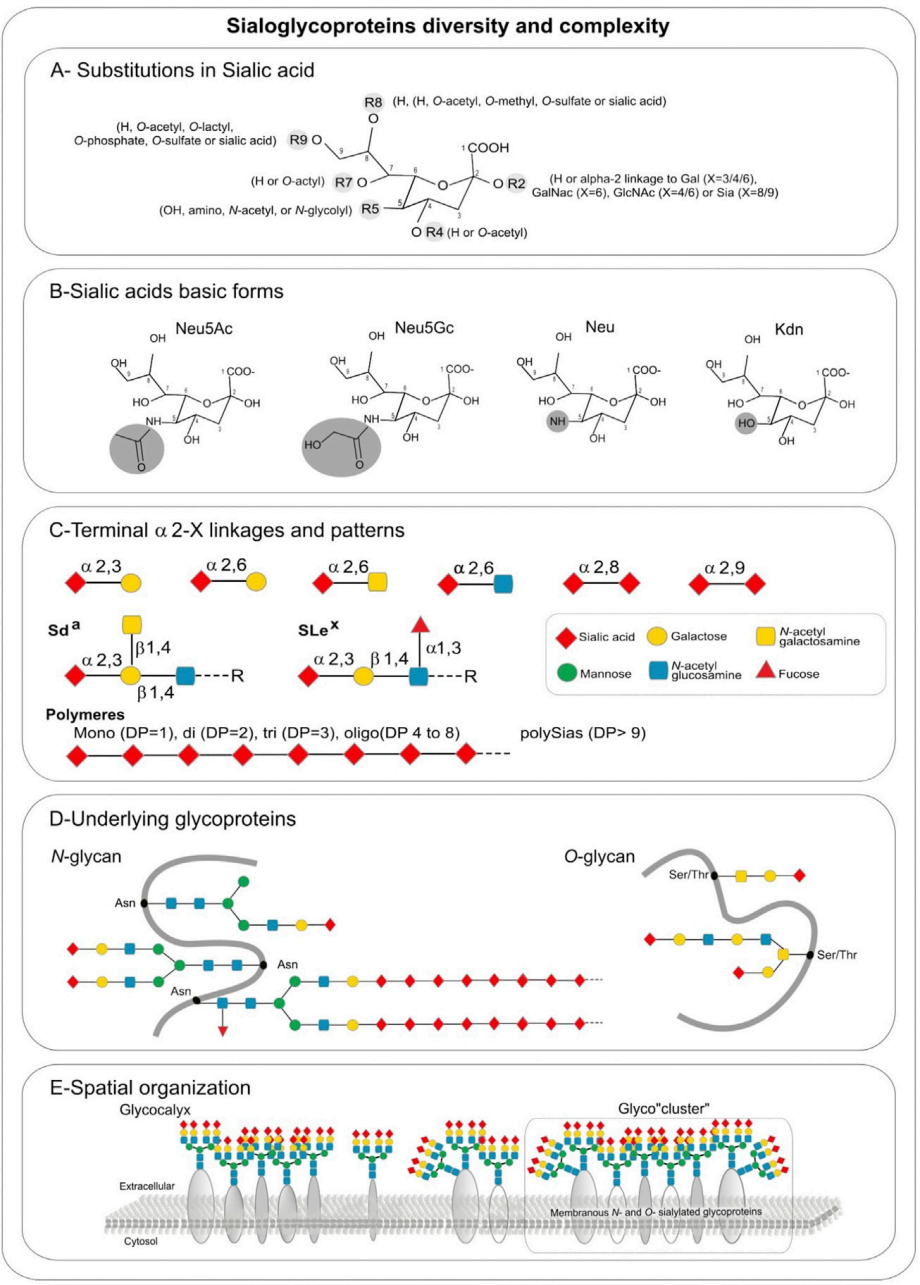
Sialic acids hierarchical complexity in sialoglycoproteins: **(A)** Substitutions found in sialic acid by various types of glycosidic linkages: in R2 an H is present in free sialic acid, or an alpha-2,X linkage to Gal (X = 3/4/6), GalNAc (X = 6), GlcNAc (X = 4/6) or Sia (X = 8/9), R4 and R7 are H or *O*-acetyl, R5 is hydroxyl, amino, *N*-acetyl, or *N*-glycolyl, R8 is an H, *O*-acetyl, *O*-methyl, *O*-sulfate or sialic acid, R9 is an H, *O*-acetyl, *O*-lactyl, *O*-phosphate, *O*-sulfate or sialic acid. **(B)** Sialic acids basic forms Neu5Ac, Neu5Gc, Neu, and Kdn. **(C)** Terminal alpha 2, X-linkages and patterns. **(D)** Underlying glycoproteins. **(E)** Spatial organization.

A first level of diversification of Sia molecules is due to various substitutions with acetyl, glycolyl, methyl, lactyl, phosphate, or sulfate groups found on Sias hydroxyl or amino groups (Figure 1A) leading to over 50 structurally different Sia derivatives in nature (Schauer, 1982). The most abundant members of the Sia family are Neu5Ac and Neu5Gc (5-*N*-glycolylneuraminic acid), followed by Kdn and Neu (neuraminic acid) (Figure 1B). However, there are marked differences in the distribution of these Sias in nature and some of these Sias completely disappear from a lineage as for Neu5Gc in human and platypus (Schauer et al., 2009; Chen and Varki, 2010). Although the biological significance of these modified Sia residues is still mostly unknown, it is interesting to note that the most Sia diversity is encountered in invertebrate deuterostomes such as echinoderms (sea urchin and starfish) and the simplest profiles are found in human (Gagneux et al., 2017). The natural occurrence of *O*-acetylated Sias is more frequently documented than other modified Sias and *O*-acetylation at C-4, C-7, or C-9 of Sias has been observed in bacteria and eukaryotes including humans (Angata and Varki, 2002; Nguyen et al., 2020). A neuraminic acid molecule may contain one to three *O*-acetyl residues, but *N*-acetyl-9-*O*-acetylneuraminic acid (Neu5,9Ac2) is the predominant form (Schauer and Kamerling, 2011). As for *O*-sulfated Sia (Sias) structures, 8-*O*-sulfation of Neu5Ac and Neu5Gc have been described in sea urchin gametes where they are involved in sperm–egg interaction and in the regulation of sperm motility, but so far almost nothing is known about the occurrence of Sias in vertebrates (Ertunc et al., 2020).

Additional levels of diversity arise from 1) the type of glycosidic α-linkage through which sialic acid moieties are attached to other glycan units, 2) the underlying sugar chains and 3) the type of glycan (Cohen and Varki, 2010). Sias can be linked in α2,3- and in α2,6-linkage to galactose (Gal)-containing glycans or in α2,6-linkage to *N*-acetylgalactosamine (GalNAc) and *N*-acetylglucosamine (GlcNAc) residues (Figure 1C). These Sias occur as monosialyl residues at the nonreducing terminal ends of glycan chains of either *N*- or *O*-glycoproteins and glycolipids. A very unique feature of Sias is that they can be linked to each other through α2,8 or α2,9-linkages forming oligo- and polySia chains found on a few glycoproteins that are called disialic acid (diSia), trisialic acid (triSia), oligosialic acid (oligoSia), and polysialic acid (polySia) according to their degree of polymerization (DP) (Troy, 1992; Sato, 2013; Sato and Kitajima, 2013). These combinations of Sia structures, modifications, DP, linkages, underlying glycans and glycan types lead to a huge diversity of sialoglycans and sialoglyconjugates constituting the so-called sialome (Varki, 2017; Sato and Kitajima 2021).

A final layer of complexity stems from the various glycosylation sites within a given protein (macroheterogeneity and microheterogeneity), and the three-dimensional organization of sialoglycoconjugates on cell surfaces (Figure 1D). Sialoglycoproteins and gangliosides can assemble into micro-domains on cell surfaces leading to the formation of glyco-cluster (Figure 1E) that could mediate differential biological functional events.

### 2.2 Sialylation machinery

**FIGURE 2:**
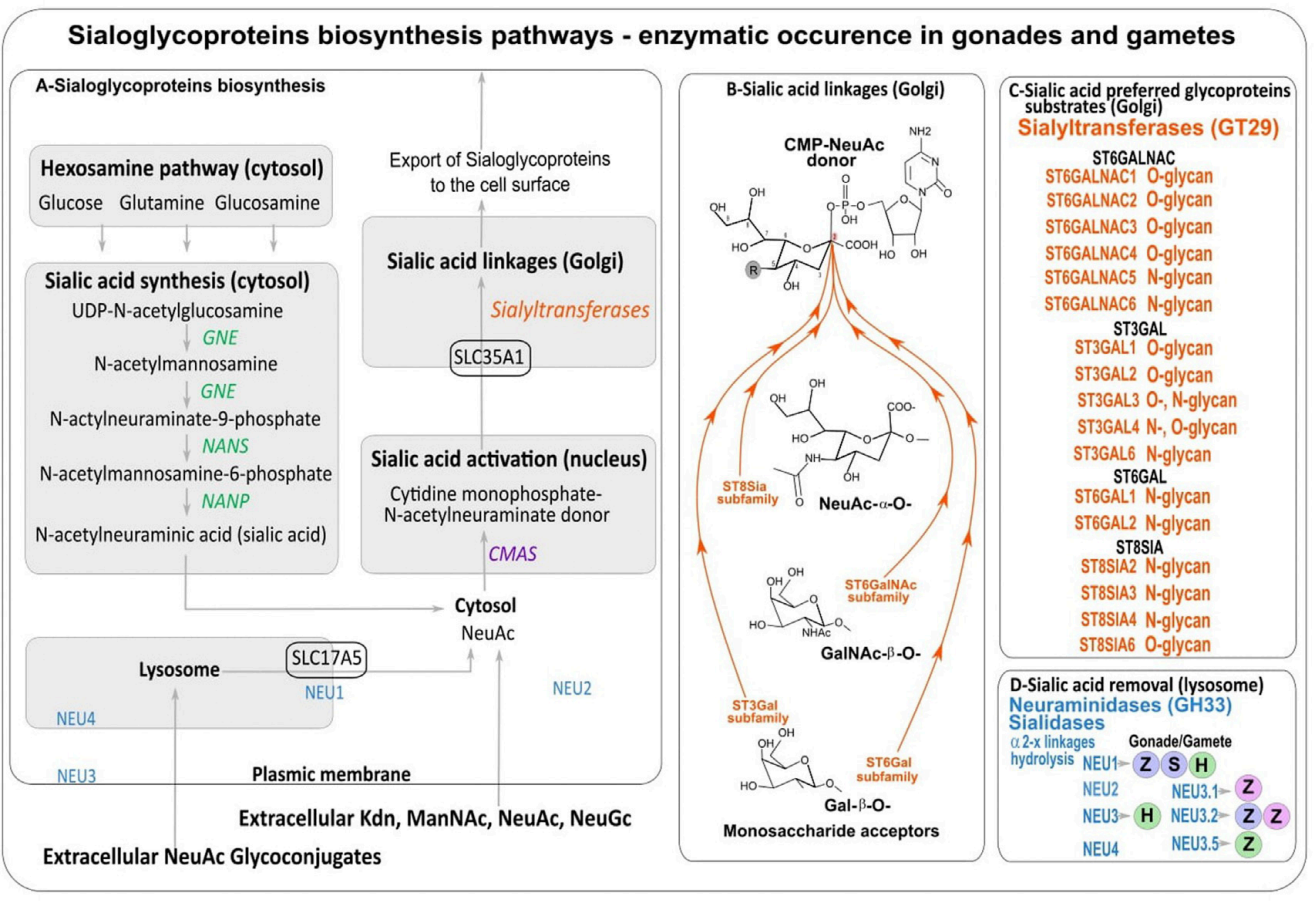
**(A)** Sialic acids biosynthesis. **(B)** Sialic acid linkages performed by sialyltransferases. **(C)** Sialyltransferases with preferred glycoproteins substrates. **(D)** Counteracting neuraminidases (NEU) detected in male (blue dots) and female gonads (pink dots) and spermatozoa (green dots) of human (H) (Lassalle and Testart, 1994; Ma et al., 2012), and fishes: zebrafish (Z) (Harduin-Lepers et al., 2008; Petit et al., 2018; Yamakawa et al., 2018) and sturgeon (S) (Li et al., 2017).

Metabolism of Sias and sialoglycoproteins schematized in Figure 2A divides into several steps diversely localized in the animal cell and a recent phylogenetic analysis reported the double origin of Sias in eukaryotic cells (Petit et al., 2018). An extrinsic Sias source in Metazoa is likely ensured by an intake of Sias from the extracellular space *via* the lysosomal SLC17A5 transporter also known as sialin associated with neuraminidase 1 (NEU1). The canonical biosynthetic pathway of Sias takes place within the cytosolic compartment of vertebrate cells (Figure 2A). Sias formation is catalyzed by a bifunctional enzyme, glucosamine UDP-GlcNAc-2-epimerase/*N*-acetylmannosamine kinase (encoded by the human GNE gene), which converts UDP-GlcNAc (uridine diphosphate N-acetylglucosamine) to ManNAc-6-P (*N*-acetyl- mannosamine 6-phosphate) and UDP (uridine diphosphate). Then, ManNAc-6-P is condensed with phosphoenolpyruvate (PEP) by the Neu5Ac-9-P synthase (NANS) forming *N*-acetylneuraminate 9-phosphate (Neu5Ac-9-P). Neu5Ac-9-P is then dephosphorylated by the cytosolic *N*-acetylneuraminate-9-phosphatase (NANP), resulting in the release of Neu5Ac to the cytoplasm. These two enzymes are widely distributed in most eukaryotic lineages (Petit et al., 2018). In prokaryotes, the condensation of ManNAc (*N*-acetylmannosamine) and PEP is catalyzed by an aldolase. The same pathway can use Man-6-P instead of ManNAc-6-P leading to Kdn.

Subsequent use of Sias depends on their activation to the sugar-nucleotide cytidine monophosphate sialic acid (CMP-sialic acid or CMP-Sia) which is catalyzed by the cytidine monophosphate *N*-acetylneuraminic acid synthetase or CMP-sialic acid synthetase (CMAS or CSS) in the nucleus of most vertebrate cells (Figure 2A). In Metazoa, orthologous sequences of human CMAS gene were retrieved in the Cnidaria, deuterostomes, and Arthropods (Di et al., 2017; Petit et al., 2018). Interestingly, in the zebrafish *Danio rerio*, there are two CSS with different substrate specificity and spatial expression one localizing in the nucleus and showing higher specificity towards Neu5Ac and the other localizing to the cytosol and showing higher specificity towards Kdn (Schaper et al., 2012).

Neu5Ac can be transformed into Neu5Gc by hydroxylation of the methyl group in the *N*-acetyl moiety of the activated CMP-Neu5Ac donor through a reaction catalyzed by cytidine monophosphate-*N*-acetylneuraminic acid hydroxylase (CMAH) (Shaw and Schauer, 1988). Orthologues of the CMAH gene were found in Porifera (Kapli et al., 2021), and chordates including tunicates, lancelets, reptiles, amphibians, most fish, and a number of mammals (Peri et al., 2018). Only a few deuterostome lineages do not have a functional CMAH gene as reported in humans, New World monkeys, ferrets, several bats, the platypus, the perciform fish *lates calcarifer* (Simakov et al., 2015; Peri et al., 2018), Sauropsids (birds and reptiles), Pinnipedia, and members of Musteloidia (Altman and Gagneux, 2019) that lack Neu5Gc.

The various activated sugar-nucleotide CMP-Sias are then transported from the cytosol to the sialylation site within the Golgi lumen (Lepers et al., 1989; Lepers et al., 1990) by the SLC35A1 antiporter (Figure 2A). SLC35A1-like sequences are found in deuterostomes from cephalochordates to human and in protists further suggesting that it is ancestral in eukaryotes (Petit et al., 2018).

**FIGURE 3:**
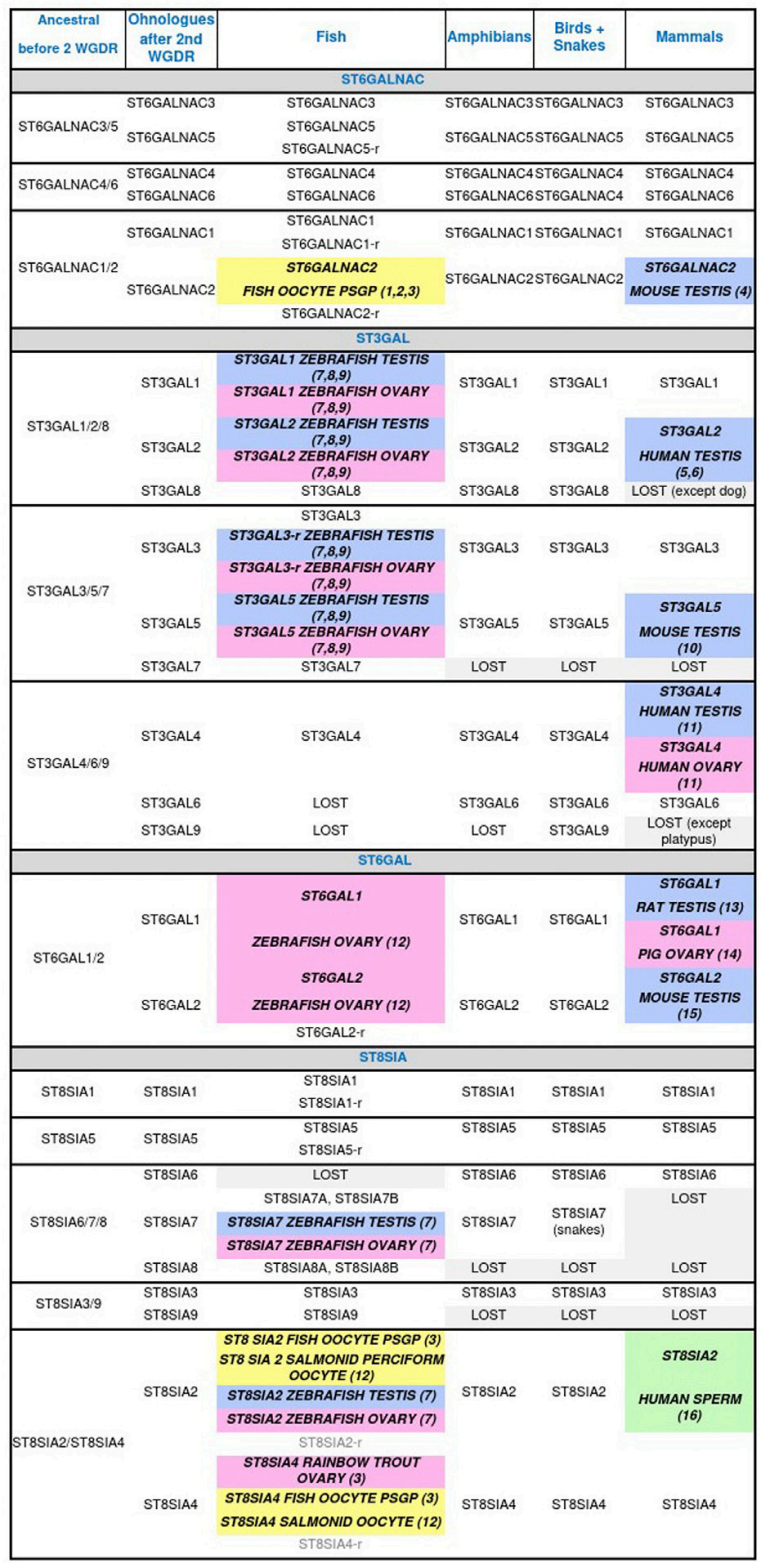
Animal sialyltransferases genes, transcripts, and/or proteins detected in male (blue) and female (pink) gonads, and male (green) and female (yellow) gametes. (1) Asahina et al., 2004 (T); (2) Asahina et al., 2006 (T); (3) Venuto M. T. et al., 2020 (G, T); (4) Kurosawa et al., 1996 (G, T); (5) Krzewinski-Recchi et al., 2003 (G, T); (6) Takashima et al., 2002 (G, T); (7) Yamakawa et al., 2018 (P); (8) Petit et al., 2018 (G); (9) Harduin-Lepers et al., 2008 (G, T); (10) Stern et al., 2000 (G, T, P); (11) Taniguchi et al., 2003 (T); (12) Harduin-Lepers et al., 2005 (G); (13) Lei et al., 2018 (T); (14) Kimura et al., 1999 (T, P); (15) Takashima et al., 2003 (G, T); (16) Simon et al., 2013 (P). Genes (G), proteins (P), related gene (r), transcripts (T), whole genome duplication round (WGDR).

Then sialylation steps and glycosidic linkage formation are carried out by specific α2,3-, α2,6- or α2,8-sialyltransferases confined to the Golgi apparatus that belong to glycosyltransferase family 29 (GT29) according to the Carbohydrate Active EnZymes database (CAZy, http://www.cazy.org) (Lombard et al., 2014) (Figure 2B). Four large families of sialyltransferases were described in Metazoan *i.e.* the ST6GAL, ST6GALNAC, ST3GAL, and ST8SIA (Harduin-Lepers et al., 2001; Harduin-Lepers et al., 2005; Harduin-Lepers, 2010; Teppa et al., 2016). A burst of novelties was acquired in each family after the two rounds of whole genome duplication (WGD) at the root of vertebrates leading to several subfamilies that were maintained or lost during vertebrate evolution (Figure 3) (Harduin-Lepers et al., 2008; Petit et al., 2010; Petit et al., 2015). An interesting case study is the one of teleost ST8SIAs that shows an extended repertoire in fish mostly shaped by the WGD events and a particular distribution in fish species (Chang et al., 2019; Venuto M. T. et al., 2020). Moreover, sequence-based analysis suggested that molecular evolution of polysialyltransferase-related sequences could account for the extraordinary diversity in polySias encountered in fish (Venuto M. T. et al., 2020). Additional modifications of Sias like *O*-acetylation are thought to take place in the Golgi apparatus and involve intraluminal *O*-acetyltransferase and acetyl-CoA transporter proteins (Figures 2B,C).

Sialoglycoconjugates exported to the cell surface function as ligands for sialic acid-binding proteins including selectins, a class of calcium-dependent C-type lectins, and Siglecs. Siglecs are Sia binding immunoglobulin (Ig)-like lectins that belong to the immunoglobulin superfamily and act as transmembrane cell surface immune regulatory receptors predominantly found on hematopoietic cells including B and T cells, natural killer cells, dendritic cells, granulocytes as well as mast cells and macrophages (Varki and Angata, 2006; Crocker et al., 2007). At least 15 human Siglecs are known to be expressed in human tissues which include CD33-related Siglecs and Siglec-1 (Sialoadhesin), Siglec-2 (CD22), Siglec-4 (myelin-associated glycoprotein, MAG), and Siglec-15. Siglec-1, Siglec-2, Siglec-4, and Siglec-15 constitute a group of highly conserved and ancient molecules which could be found in the common ancestor of vertebrates (Bornhöfft et al., 2018). The remaining Siglecs including the CD33-related Siglecs show marked inter-species differences in repertoire (a loss of Siglecs genes was reported in rodent), sequence, and binding preference (Angata, 2006).

Finally, sialidases, also known as neuraminidases (NEU genes) release α-linked Sias from glycoconjugates and polysaccharides. These enzymes are common in Metazoa and also in microorganisms. The human enzymes NEU1, NEU2, NEU3 and NEU4 are classified in the glycoside hydrolase family GH33 of the CAZy classification (Henrissat and Bairoch, 1996). They are specific of sialylated glycoconjugates and show characteristic tissue and cell expression pattern (Monti et al., 2010). Interestingly, NEU1 is found in the lysosome of Metazoa where it is involved in the hydrolysis of exogenous sialyloglycoconjugates (Figure 2D) and recent phylogenetic analyses described NEU1 as the most ancient neuraminidase whereas the clade NEU2/NEU3/NEU4 would have another origin in deuterostomes (Giaccopuzzi et al., 2012; Petit et al., 2018).

## 3 Oocyte Sias diversity and their multiple roles

**FIGURE 4:**
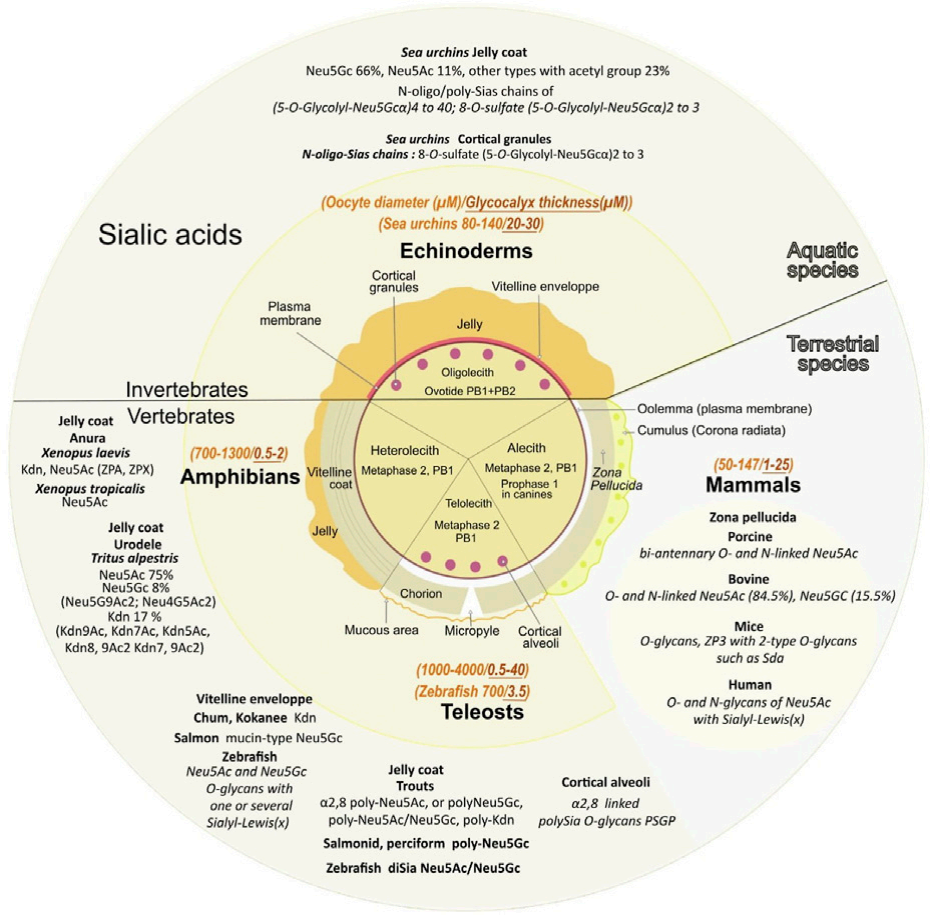
Various sialic acids and glycoproteins present in mature oocyte coat from diverse species. The central circle depicts the specific envelopes surrounding each type of mature oocytes also called “unfertilized eggs”. Oocytes have not completed their meiosis until fertilization except for echinoderms, the oocyte displays two polar bodies (PB1+PB2). Oocytes from amphibians, mammals and teleosts are arrested in the metaphase of the second meiotic division after the expulsion of one polar body (PB1). Canine oocytes are arrested in the prophase of the first meiosis division. Except for alecith (poor in yolk) mammals, oocytes from other species depicted contain vitelline reserves. Note that the vitelline region contains multiple layers in amphibians. Mature oocyte sizes compared to their extracellular matrix are respectively reported in italic orange and italic brown (yellow middle circle). Major sialic acid structures present in the outer coat of gametes and involved internal or external fertilization are mentioned for aquatic and terrestrial species (outer circle).

Deuterostome oocytes are surrounded by a soft gelatinous vitelline envelope and an important jelly coat in echinoderms and amphibians, a thick chorionic membrane in fishes, or a *zona pellucida* in mammals, all composed of numerous different carbohydrates including a high diversity of sialylated glycoproteins (Monné and Jovine, 2011). In a single phylum, the composition of Sias varies among the different species (Figure 4).

### 3.1 Oocytes from marine and freshwater species

#### 3.1.1 A high content and a variety of sialic acid derivatives surround oocytes

The surrounding transparent gelatinous layer or jelly coat of sea urchin oocytes (up to 30 μM) is rich in polysaccharides and contains unique sialylated glycoconjugates (Kitazume et al., 1994; Kitazume et al., 1996). Mass spectral analyses determined that Neu5Gc is the major Sia while Neu5Ac is the second most common (respectively 66% and 11%) and 23% of other Sias including Neu5GcS, Neu5Gc9Ac, Neu5AcS, Neu5Gc7,9Ac2, Neu5,9Ac2, Neu5Gc8Ac, Neu5Gc7Ac, Neu5,7Ac2, Neu5Gc8,9Ac2, and Neu5,8Ac2 (Yeşilyurt et al., 2015) (Figure 4).

Amphibian oocytes thick jelly coat contains several layers: three layers in the Anurans *Xenopus laevis* and six in *Rana pipiens* (*Lithobates pipiens*, Taxonomy ID: 8404). In the pipidiae *X laevis,* oocytes Sias are localized at the interface between the first and second layers, charged by oviductal Sias secretions as they go through the oviduct (Mozingo and Hedrick, 1999). The jelly coat contains four types of sialylated glycans with three of them containing Kdn and one Neu5Ac (Plancke et al., 1995), with sulfated (Strecker et al., 1995), or doubly sulfated anionic glycans (Guerardel et al., 2000). An alteration of the Sias content in the jelly coat leads to inhibition of sperm penetration (Gurushankara et al., 2012). In *Xenopus tropicalis* jelly coat, among the 19 anionic glycans detected, six contain Neu5Ac while *Xenopus borealis* has no detectable anionic glycans and exclusively contains neutral *O*-glycans (Li et al., 2011). In the urodele *Triturus alpestris* (*Ichthyosaura alpestris*, Taxonomy ID: 54263) (Florea et al., 2006) Neu5Ac, Neu5Gc, and Kdn derivatives (respectively in the ratio 75, 8, 17) are detected with a high degree of *O*-acetylation of Neu5Ac and Kdn: Neu5,9Ac2 represents 56% of Neu5Ac derivatives (traces of Neu4,5Ac2 were also observed), Kdn represented 42% of total Kdn derivatives with mono-*O*-acetylated (39% Kdn9Ac, 6% Kdn7Ac, 2% Kdn5Ac). In the vitelline envelope, among the six ZP glycoproteins also found in other species, ZPA/ZP2/gp69/64, ZPB/ZP4, ZPC/ZP3/gp41, ZPD, ZPX, and ZPY (Hedrick, 2008), only ZPA (Vo et al., 2003) and ZPX are sialylated (Barisone et al., 2003). ZPA displays 30% of the sperm binding activity, while the rest is ascribed to ZPC (Miwa, 2015). It has to be noted that ZPB and ZPC are found in all species, but ZPA is not found in teleost fish, and ZPX is mainly found in invertebrates but is not present in mammals (Feng et al., 2018). In toad *Bufo arenarum* (*Rhinella arenarum*, Taxonomy ID: 38577), Sias composition varies with high levels of Sias present in the preovulation period associated with an 80% decrease in the post-ovulation period, associated with changes in neuraminidases and sialyltransferases activity (de Martinez et al., 1975).

In teleosts, high content of Sias is present around the oocytes, in the *zona radiata* (the equivalent of the mammals ZP) of the dusky grouper *Epinephelus marginatus* (Mandich et al., 2002), the swordfish *Xiphias gladius* (Ortiz-Delgado et al., 2008), the bluefin tuna *Thunnus thynnus* (Sarasquete et al., 2002), and the trout (Kitazume et al., 1994). Kdn is the major component of the vitelline envelope of the chum *Oncorhynchus keta* and kokanee salmon *Oncorhynchus nerka* while cherry salmon *Oncorhynchus masou* contains an analogous family of mucin-type glycoproteins with ending Neu5Gc as the major component (Inoue and Inoue 1997; Gallo and Costantini, 2012). In rainbow trout *Oncorhynchus mykiss*, the vitelline envelope Kdn-glycoprotein is the unique polysialoglycoprotein (PSGP). In *D. rerio*, *N*- and *O*-glycans are predominantly bearing a single terminal Neu5Ac or Neu5Gc residue but disialylated structures exist. Monosialylated Neu5Ac and Neu5Gc and Neu5Ac-Neu5Gc or Neu5Gc-Neu5Gc disialyl unit extends from an internal Gal of in internal Lewis(x) (Le^x^) structure (Neu5Acα2,3Galβ1,4(Fucα1,3)GlcNAc) (Guérardel et al., 2006).

#### 3.1.2 Unique oocytes species-specific polysialylation contributes to sperm acrosomal reaction

Unlike other monosaccharides, Sias polymers with various degrees of polymerization (DP) are evolutionarily conserved from sea urchin to humans (Sato and Kitajima, 2013). Sea urchin and fish oocytes display a variety of different Sias polymers in contrast to mammals that mainly contain α2,8-linked *N*-acetylneuraminic acid residues on a restricted number of polysialylated proteins. In mammals, only a few glycoproteins are polysialylated including: the neural cell adhesion molecule (NCAM) in the central nervous system (Schnaar et al., 2014), synaptic cell adhesion molecule (SynCAM 1), neuropilin-2 (NRP-2), the C–C chemokine receptor type 7 (CCR7), E-selectin ligand-1 (GLG1), the α subunit of the voltage-dependent sodium channel, the CD36 scavenger receptor in human milk, and polysialyltransferases themselves (James and Agnew, 1987; Close and Colley, 1998; Yabe et al., 2003; Galuska et al., 2010; Mühlenhoff et al., 2013; Kiermaier et al., 2016; Werneburg et al., 2016). PolySias are long negatively charged and linear polymers that form a strong repulsive field (Yang et al., 1994).

In sea urchins oocytes, the Sias-rich polysaccharides are composed of Neu5Gc (Valle et al., 2015). In *Hemicentrotus pulcherrimus* oocytes, polySias chains (5-*O*-glycolyl-Neu5Gcα2)n have a DP (n) ranging from 4 to 40 Neu5Gc residues, and these polyNeu5Gc glycans are *O*-glycosidically linked to threonine residues (Kitazume et al., 1994; Yeşilyurt et al., 2015). The *S. purpuratus* oocyte jelly coat contains a PSGP with 25 polySias chains *O*-linked to both threonine (two-thirds) and serine (one-third) residues (Kitazume et al., 1994). These polySias in the oocyte jelly are highly specific to each species of echinoderm and initiate the sperm acrosomal process through calcium entry into the sperm head. The acrosomal reaction that causes granules exocytosis and release of proteolytic enzymes digests part of the thick protective jelly coat to allow access to the oocyte surface. The oocyte jelly also possesses 8-*O*-sulphated (5-*O*-glycolyl Neu5Gcα2)_n_ with n ranging from two to 3 at the nonreducing end of the oligo/polySia chain found on the 350 kDa sperm receptor on the oocyte membrane (Kitazume et al., 1996; Sato and Kitajima, 2013), and Neu5Gc8S (Schauer, 2012) (Figure 4).

PolySias structures on glycoproteins were first discovered among vertebrates in rainbow trout eggs and isolated from salmonid as polyNeu5Gc (Inoue and Iwasaki, 1978; Kitajima et al., 1986). These teleost fishes polySias are commonly observed with a remarkable degree of structural diversity in the α2,8-linked polySia chains. Homopolymers of α2,8-linked Neu5Ac or Neu5Gc and heteropolymers containing both Neu5Ac and Neu5Gc residues, or Kdn and *O*-acetyl substitution at C4, C7, C9, or *O*-lactyl substitution at C9 were described (Kitajima et al., 1986; Sato et al., 1993). These chains are differentially expressed in various fish species. PolyNeu5Ac and polyNeu5Gc are present in the lake trout *Salvelinus namaycush*, poly (Kdn) in the rainbow trout *O. mykiss*, polyNeu5Aca/Neu5Gc in the brook trout *Salvelinus fontinalis*, polyNeu5Ac/Neu5Gc in the brown trout *Salmo trutta fario* and the Japanese char *Salvelinus leucomaenis pluvius*, polyNeu5Gc in the chum salmon *O. keta*, the cherry salmon *O. masou ishikawai*, the perciform fish *Sander lucioperca* and the salmonid fish *Coregonus maraena* (Venuto M. T. et al., 2020; Venuto M. T. et al., 2020). Among Osteichtyes superclass, only one polysialyltransferase is detected in perciforms (ST8SIA2), and three are present in salmonids (ST8SIA2-r1, ST8SIA2-r2, and ST8SIA4) (Venuto M. T. et al., 2020; Venuto M. T. et al., 2020)). In zebrafish eggs, diSia (Neu5Ac/Neu5Gc) is identified in *N*- and *O*-glycans and is finely regulated during embryonic development (Guérardel et al., 2006; Chang et al., 2009).

#### 3.1.3 PolySias stored in oocytes cortical granules counteract polyspermy and pathogens

During oogenesis in most animal species, mature oocytes accumulate large amounts of glycoconjugates including sialylated glycans in intracellular secretory organelles derived from the Golgi, the so-called cortical granules/vesicles, localized in the peripheral cytoplasm (Gallo and Costantini, 2012).

Sea urchins cortical granules localized in the cortex beneath the vitelline envelope, contain Neu5Ac8S (Yamakawa et al., 2007). These granules are exocytosed at fertilization and contribute to the destruction of the sperm binding capacity of the vitelline layer. Mainly the release of trypsin-like proteases results into the vitelline layer hardening to form a barrier against polyspermy by peroxidases catalyzing tri-tyrosine crosslink.

The teleost unfertilized oocyte cortical granules, named cortical alveoli, are filled with polySias with a high molecular weight (200 kDa) and degrading enzymes. During the cortical reaction after the monospermic fertilization, cortical alveoli exocytosis into the perivitelline space results in polySias cleavage to low-molecular-weight units of around 9 kDa. The cortical alveoli content prevents polyspermy and blocks the micropyle opening in the thick chorion surrounding the oocyte through which the first spermatozoa entered at fertilization. The cortical alveoli glycoproteins are subdivided into two groups, the α2,8-.linked polySia group-containing polyanionic glycan units attached to protein through *O*-glycosidic linkages and the other containing bulky multiantennary *N*-linked glycans named hyosophorin (Taguchi et al., 1994). The PSGP (polysialoglycoprotein) polySias display species-specific structural diversity in fish. In rainbow trout *Salmo irideus* (Inoue and Iwasaki, 1978), salmon *S. leucomaenis pluvius* (Iwasaki and Inoue, 1985), and flounder *Paralichthys olivaceus* (Seko et al., 1989) the PSGP from the hyosophorin group are the major constituent of cortical alveoli. Rainbow trout *Salmo gairdneri* (*Oncorhynchus mykiss gairdneri* Taxonomy ID: 857570) oocyte PSGP occur with α2,8-linked polysialic acid chains linked to *O*-glycosidic carbohydrate units with a length up to 25 sialic acid residues (Kitajima et al., 1986), and are capped at their non-reducing ends by Kdn (Nadano et al., 1986). In the rainbow trout *S. gairdneri*, PSGP Sias are Neu5Gc, while in *Salmo* and *Salvelinus* species they contain both Neu5Ac and Neu5Gc (Kitajima et al., 1986; Seko et al., 1989). In Indian medaka *Oryzias latipes* and *Fundulus heteroclitus,* a tetraantennary sialoglycan unit is present (Taguchi et al., 1993; Taguchi et al., 1996). PSGP biosynthesis has been reported at late stages during oogenesis (Asahina et al., 2004; Asahina et al., 2006). The sialylation of PSGP is catalyzed by at least four sialyltransferases present inside the oocyte cortical granules and acting synergistically: an α2,6-sialyltransferase (ST6GALNAC), and three α2,8-sialyltransferases (ST8SIA2-r1, ST8SIA2-r2, and ST8SIA4) (Asahina et al., 2004; Asahina et al., 2006; Venuto M. T. et al., 2020). PolySias on PSGP also serve to block polyspermy and protect the oocyte from pathogens in the extracellular environmental medium. The highly modified polySias groups of PSGP, displaying *O*-acetylation of the hydroxyl-groups on C4, C7, C9, and Kdn at the non-reducing termini, confer resistance to bacterial sialidases. PolySias on PSGP have also been shown to be a Ca^2+^ and ionic storage reservoir and have been supposed to be involved in osmoregulation during early development (Shimoda et al., 1994).

### 3.2 *Zona pellucida, Sias, and* sialoglycoproteins in other vertebrates

Oocytes from non-aquatic vertebrates do not bear PolySias (Simon et al., 2013) but display unique Sias structures compared to aquatic species localized in the vitelline envelope and the *zona pellucida.* In addition, humans and several mammalian species lack Neu5Gc (Altman and Gagneux, 2019) due to a single exon deletion/mutation in the gene encoding CMAH (Varki, 2017) (Figure 4). The loss of vertebrate-specific Neu5Gc is puzzling and evolutionary scenarios include the proposal for a strong selection by various parasites, but also a selection *via* females towards paternal Neu5Gc. Humans also have circulating antibodies specifically targeting Neu5Gc which is immunogenic and hypothesized to lead to chronic inflammation and other pathologies (Padler-Karavani et al., 2008). Neu5Gc antibodies do not seem to have a biological function in gametes and reproduction (Altman and Gagneux, 2019), but it cannot be excluded that they could play a role in the female genital tract to avoid interspecies fertilization.

The transparent extracellular ZP surrounding oocytes of non-aquatic vertebrate species is a viscous fibrogranular structure at the border between the oolemma plasma membrane and the inner layer of follicle cells of the cumulus matrix (*corona radiata*) (Sinowatz et al., 2001). ZP matrix proteins have been described in granulosa cells and are supposedly involved in folliculogenesis (ZP3 in rabbit, mice, and human, and both ZP3 and ZP1 in bovine and porcine (Prasad et al., 2000; Hasegawa et al., 2007; Gook et al., 2008). ZP is also involved in the protection against physical damages as it is a thick elastic matrix composed of ZP proteins assembled into polymers, which hardens upon fertilization after a series of changes that also provide a polyspermy block (Papi et al., 2010; Monné and Jovine, 2011; Fahrenkamp et al., 2020; Wassarman and Litscher, 2021). In some mammals, ZP proteins have been demonstrated to be involved in species-specific recognition and binding (mouse and human ZP2) to the spermatozoa (Avella et al., 2014; Bhakta et al., 2019), and probably play a role in the ZP matrix modification associated to the polyspermy block (mouse and human ZP1 crosslinker of ZP filaments (Greve and Wassarman, 1985; Nishimura et al., 2019). The composition of ZP proteins varies among species. In mammals, ZP is composed of three to eight proteins. In most mammals including humans, ZP is composed of four proteins: ZP1, ZP2/ZPA/gp69/64, ZP3/ZPC/gp41, and ZP4/ZPB, while in mice it is composed of ZP1, ZP2, and ZP3, and in cows, dogs, dolphins, and pigs, ZP2, ZP3, and ZP4. Marsupials from South America have ZP1, ZP2, ZP3-1b, and ZP3-1c or from Australia ZP1, ZP2, ZP3-1a, ZP3-1b, ZP3-1c, ZP4, ZPAX, while monotremes contain ZPY, ZP1 ZP2, ZP3-1a, ZP3-1b, ZP3-2, ZP4, ZPAX (Wassarman and Mortillo, 1991; Lefièvre et al., 2004; Moros-Nicolás et al., 2021). ZP is also composed of acidic glycoproteins with *N*-linked and *O*-linked carbohydrate chains and various sialylated oligosaccharides (Noguchi et al., 1992; Nakano and Yonezawa, 2001; Dell et al., 2003; Pang et al., 2007; Velasquez et al., 2007), (Figure 4). In mammals, sialylated ZP has also been proposed to act in immune recognition as oocytes lack major histocompatibility (MHC) class I molecules that mediate self-recognition, which makes them vulnerable to natural killer cells (Clark, 2010).

In Birds, ZP1, ZP3, and ZPD are the major egg-coat ZP proteins (Nishio et al., 2018). In chicken, ZP2 is concentrated around the germinal disk region (Nishio et al., 2014; Nishio et al., 2018. Mutation of the T168 *O*-glycosylated site reduces the sperm-binding activity of recombinant chicken ZP3 (Han et al., 2010). The highest concentration of Sias is present in the vitelline membrane, but Sias are also found in chalaza, yolk and white (Nakano et al., 1994).

Chimeric engineered mice that express human ZP3 and acquire mouse-type *O*-linked glycans deprived of SLe^x^ structures are capable to bind murine but not human sperm (Dell et al., 2003; Gahlay et al., 2010). In addition, murine oocytes ZP3 deficient in core 1-derived *O*-glycans (mutated on T-synthase) undergo fertilization (Williams et al., 2007). Mutated mouse oocytes lacking *N*-glycans (mutated for Mgat1, encoding *N*-acetylglucosaminyltransferase I) (Shi et al., 2004), or deficient in core 2-derived *O*-glycans support mouse sperm binding but not human sperm binding compared to wild-type mouse oocytes suggesting a glycan-independent process, and other determinants necessary for taxon-specificity (Hoodbhoy et al., 2005). The respective role of the different oligosaccharide ligands remains to be elucidated as the majority of the *O*-glycans linked to murine ZP3 are sialylated including core two *O*-glycans, LacNAc (Galβ1-4GlcNAc), LacdiNAc (GalNAcβ1-4GlcNAc), Galα1-3Gal, and Neu5Acα2-3(GalNAcβ1-4)Galβ1,4GlcNAc (Sd^a^ antigen), and SLe^x^ (Dell et al., 2003; Dall'Olio et al., 2014).

In porcine, the major *O*-linked and *N*-linked oligosaccharides found in the ZP matrix are bi-antennary complex type glycans with antennae containing sialylated polyLacNAc sequences (Dell et al., 1999; Nakano and Yonezawa, 2001). Glycoprotein ZP2 display six, ZP3 three, and ZP4 five potential *N*-glycosylation sites, while ZP4 contains three and ZP3 six potential *O*-glycosylation sites (Yurewicz et al., 1991; Yonezawa et al., 1997). *N*-glycosylation of porcine ZP glycoproteins seems crucial in sperm penetration and binding to the ZP (Lay et al., 2013). While the role of sialylated glycoproteins was not established, *N*-glycosylation of ZP3 might increase sperm binding (Lay et al., 2011). Pig Sias are shown to be synthesized by α2,6-sialyltransferases (Kimura et al., 1999).

In bovine, 77% of the carbohydrate chains of ZP glycoproteins are acidic chains containing Sias (Katsumata, 1996), with mainly Neu5Ac (84.5%) and Neu5Gc (15.5%) linked to Galβ1-4GlcNAc and GalNAc with α2,3- and α2,6-linkages, on *N*- and *O*-glycans respectively (Velasquez et al., 2007). A ZP3/ZP4 association mediates interaction with the spermatozoa and depends on *N*-glycans (Suzuki et al., 2015), with ZP4 having the strongest sperm-binding activity (Yonezawa et al., 2001). The N-glycosylation of ZP3 (Suzuki et al., 2015), and the Sias sequence of Neu5Ac (α2-3)Gal (β1-4)GlcNAc (Velasquez et al., 2007) are also implicated in sperm-ZP binding.

In human, mutations in ZP genes encoding ZP1, ZP2, ZP3, and ZP4 are involved in cases of female infertility (Wassarman and Litscher, 2021; Wassarman and Litscher, 2022). Recombinant studies implying glycosylation of human ZP3 revealed this glycoprotein is essential to induce acrosomal exocytosis but not for sperm binding (Chakravarty et al., 2008). Human oocytes ZP *N*- and *O*-glycans display unique SLe^x^ structures that were initially proposed to act in the sperm interaction with the oocyte but later experiments showed they were dispensable (Miranda et al., 1997; Oehninger et al., 1998; Pang et al., 2011; Wassarman, 2011; Clark, 2013; Gupta, 2018; Bhakta et al., 2019; Gupta, 2021; Tumova et al., 2021). ZP mutated mouse oocytes rescued by human ZP2, and deprived of SLe^x^ in their zona pellucida matrix were shown to bind to human sperm, and ZP2 sperm-binding domain was determined to be required for human sperm-oocyte recognition and zona pellucida penetration (Baibakov et al., 2012; Avella et al., 2014). Transgenic mouse oocytes ZP composed of mouse ZP1, ZP3, and human ZP4 do not bind mouse sperm, and transgenic mouse ZP containing human ZP1, ZP3, and ZP4 do not bind human sperm and are infertile (Avella et al., 2014; Bhakta et al., 2019), supporting ZP2 as necessary and sufficient for human and mouse sperm binding. Genetic removal of ZP2 N-terminus glycan does not impede sperm binding or female fertility (Tokuhiro and Dean, 2018), showing that ZP2 N-terminus is dispensable for gamete recognition (Avella et al., 2014; Tokuhiro and Dean, 2018). Chimeric mouse ZP2 proteins, with a human N-terminal domain, support human sperm binding evidencing that the N-terminus of ZP2 being involved in human species-specificity binding of sperm. (Avella et al., 2014). Morevover, the human sialyltransferase ST3GAL4 gene is present in ovaries but its precise role is not understood (Tanagushi et al., 2003). ZP Sias could mediate other functions related to immune recognition (Clark, 2010). Indeed, SLe^x^ structures are ligands for Siglec nine tyrosine-based immunoreceptors that can transmit signals to a variety of different types of leukocytes and lymphocytes to allow oocyte survival and block the female immune response (Angata and Varki 2002; Avril et al., 2004).

A clue to understanding this diversity could come from the ZP glycoproteins synthesis study that differs between several vertebrate species. Mammalian ZP glycoproteins are transcribed in human, monkey, rabbit, dog, and cow both by the oocyte and the follicular cells, while in mice the oocyte exclusively contributes to their synthesis (Sinowatz et al., 2001). In birds, ZP1 is from the liver and both ZP3 and ZPD are from follicular granulosa cells (Nishio et al., 2018).

## 4 Sperm preparation through the coating/uncoating changes in Sias

**FIGURE 5:**
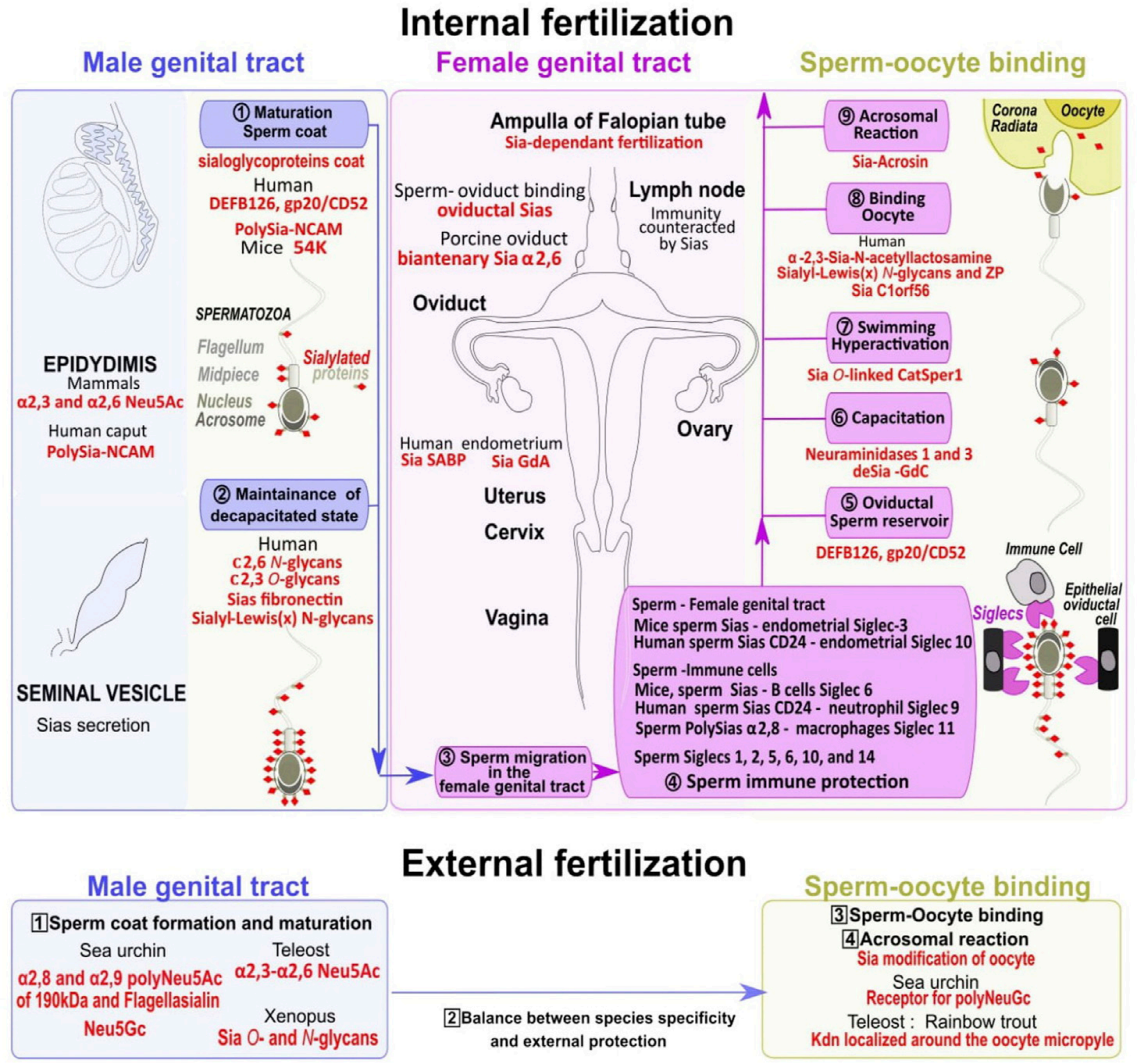
Dynamical changes in sialoglycoproteins during the sperm journey to reach the fertilized oocyte. Spermatozoa maturation first occurs in the epididymis and the seminal vesicle in the male genital tract with the addition of sialic acids to glycoconjugates (including sialoglycoproteins) in the outer coat (khaki dots surmounted by red diamonds) (1, numbers in circles). Sialoglycoproteins from the head and tail parts help to maintain spermatozoa uncapacitated state (2) (upper blue panel). In the female genital tract (upper pink panel) multiple steps allow sperm to gain mobility (3), escape immune response (4), form an oviductal reservoir (5), acquire fertility through capacitation (6) associated with high swimming potential (7), bind to the oocyte (8), and finally trigger the acrosomal reaction to remove the oocyte extracellular sialic acid coat (9) (upper yellow panel). Numerous Siglecs localized on epithelial oviductal cells and immune cells in the female genital tract are involved in spermatozoa protection/survival (4). In species with external fertilization the sperm coat is formed within the male genital tract (numbers in squares, 1, lower blue panel), a balance exist between species specificity and protection (2), and the specific oocyte sialic acid coat removal also requires an extracellular spermatozoa acrosome reaction (3, yellow panel) before fertilization (4). Several species-specific sialic acids acting at different levels of the sperm journey in both internal and external fertilization are highlighted in red.

The spermatozoa of several invertebrates such as sea urchins, and non-mammalian vertebrates including most fishes and amphibians possess the capacity to fertilize oocytes when they leave the testis after they have been released for external fertilization. On the contrary, in mammals, testicular spermatozoa do not have fertilizing capacity but need to mature by passing through the epididymis where sialic acid is deposited to cap most sperm glycan chains of the membrane cell surface. From the high numbers of spermatozoa deposited in the female reproductive tract, only a few thousand enter the oviduct, and fewer reach the ampulla region of the oviduct at the appropriate time window for fertilization (Varki, 2017). If the Sias coat regulates sperm migration through the cervical mucus, and the formation of the sperm oviductal reservoir, spermatozoa remain unable to fertilize an oocyte until sialoglycoproteins partial removal exposes specific critical structures during capacitation (Varki, 2017), (Figure 5).

### 4.1 Sias addition to sperm is a prerequisite for their maturation

#### 4.1.1 Sperm from aquatic species

The sea urchin sperm surface of *Lytechinus variegatus* is coated by highly sulfated polysaccharide and composed almost exclusively of Neu5Ac (Valle et al., 2015) that potentiate sperm acrosome reaction (Hirohashi and Vacquier, 2002). *H. pulcherrimus* and *Strongylocentrotus purpuratus* also present a majority of Neu5Gc residues (Kitazume et al., 1994; Kitazume et al., 1996; Miyata et al., 2004). The sperm flagella displays two distinct polySia structures, which are α2,8- and α2,9-linked polymers of Neu5Ac: an 8-*O*-sulfated (9Neu5Acα2)_n_ structure of 40–80 kDa (with a DP of 15) carried by flagellasialin, and an α2,8-linked polyNeu5Ac *O*-glycan of 190 kDa (Miyata et al., 2004; Miyata et al., 2006; Miyata et al., 2011). Flagellasialin is involved in voltage-sensitive sodium and calcium channels that control sperm motility (Kambara et al., 2011). The flagellum and the head express a 190 kDa glycoprotein that facilitates proteins rearrangement on the spermatozoa surface upon activation (Miyata et al., 2011). As a dynamic mosaic bilayer, the plasma membrane is enriched in microdomains (lipid rafts) that serve as platforms for protein segregation (Simons and Ikonen, 1997; Hakomori, 2004; Kim et al., 2020). Spermatozoa Sias are enriched in these membrane microdomains (Chen and Varki, 2010) where flagellasialin also tends to concentrate (Sapoń et al., 2019). Clustered patches formed in dynamic microdomains could relocate sialyloglycoproteins on the sperm plasma membrane and play an active role during capacitation (Wang et al., 2021).

In *Xenopus* spermatocytes Sias are histochemically detected in *O*-linked oligosaccharides and *N*-glycans (Valbuena et al., 2012).

In teleost such as in Siluriform catfish, *Heteropneustes fossilis,* a seminal vesicle present in the male reproductive tract (Legendre et al., 1996) ensures spermatozoa storage and secretion of Sias and could play a role in sperm survival and maintenance (Senthilkumaran and Joy, 1993). In the teleost catfish *Clarias batrachus*, Sias associated with sperm in the seminal vesicle are submitted to annual variations with high levels in the spawning phase (summer peak) and low values in the resting phase (Singh and Joy, 1999). In Acipenseriformes sturgeons, *Acipenser baerii,* and *Acipenser schrenckii,* Neu1 is a biomarker associated with sperm quality (Li et al., 2017). The concomitant expression of the teleost-specific α2,3-sialyltransferase gene *st3gal*3-r and the two sialidase genes *neu*1 and *neu*3.5 in zebrafish testis has not been explained (Yamakawa et al., 2018).

#### 4.1.2 Sperm sialome maturation and immune protection in the female genital tract of several amniotes

The mammalian sperm sialome is established during spermatogenesis in the testis. It requires afterwards an epididymal maturation and the incorporation of sialylated seminal fluid components around the sperm membrane during ejaculation. These incorporations increase the number and the diversity of the sperm Sias involved in sperm progression through the female genital tract. To limit microbial attack, the female reproductive tract is submitted to immune surveillance to promote the destruction of pathogens (Wira et al., 2011) and an uterus endometrium antimicrobial resistance (Nasu and Narahara, 2010). Sperm entry into the uterine lumen triggers an immune response (or leukocytic reaction) that destroys most of the spermatozoa (Thompson et al., 1992). To resist this massive destruction, spermatozoa (as oocytes) lack major histocompatibility (MHC) class I molecules involved in the immune system self-recognition which renders them less susceptible to class I restricted cytotoxic effect by T lymphocytes, but are vulnerable to natural killer cells (Dell et al., 2003) that spermatozoa have to bypass.

The first maturation process in the Sias coat of spermatozoa occurs in the epididymis (Holt, 1980; Hoffmann and Killian, 1981; Toowicharanont and Chulavatnatol, 1983; Ma et al., 2016). Chicken spermatozoa are extensively sialylated and contain residues of α-mannose, α-glucose, α- and β-galactose, α-fucose, α- and β-*N*-acetylgalactosamine, *N*-acetyllactosamine, and monomers and dimers of *N*-acetylglucosamine restricted to the acrosomal region (Peláez et al., 2011).

In mice, sperm bears α2,3-linked Neu5Ac, with more than 20% of Sias transferred to mature sperm in the caput and *corpus epididymis* by sialyltransferases (Ma et al., 2016). ST8SIA3, ST6GALNAC2, and ST6GAL2 are detected in mouse testis and could probably account for sialylations on sperm (Yoshida et al., 1995; Kurosawa et al., 1996; Takashima et al., 2003) or ST6GAL transcripts in rat (Bessler et al., 1995). Interestingly, in ST8SIA2 and ST8SIA4 KO mice, the loss of polySias alters the contractile phenotype of peritubular smooth muscle in the postnatal testis (Hachem et al., 2021). A 54 kDa sialoglycoprotein binds to the immature mouse sperm during maturation in the epididymis and is implicated in the protection of sperm from phagocytosis by macrophages (Toshimori et al., 1991). Sperm phagocytosis and antigenicity are also proposed to be partly avoided in chicken, mouse, and human by a similar mechanism based on Sias (Steele and Wishart, 1996; Yudin et al., 2005; Ma et al., 2016).

In primates, β−defensin 126 (DEFB126) formerly known as epididymal secretory protein 13.2 (ESP13.2) coats sperm surface until completion of capacitation (Yudin et al., 2005), and gp20/CD52 was also reported to coat the whole sperm with an elevated presence over the equatorial segment of the sperm head (Della Giovampaola et al., 2001). DEFB126 adsorbed on the sperm surface is necessary for progression through the cervical mucus until the upper oviduct, and the site of fertilization (Tollner et al., 2008; Szczykutowicz et al., 2019). DEFB126 and gp20/CD52 Sias moieties are responsible for cloaking and probably protect the sperm surface from immune recognition in the female reproductive tract (Zhou et al., 2004). Sialylation on spermatozoa (Tecle and Gagneux, 2015) provides negatively charged and repulsion surfaces between sperm and oviductal cells necessary for their transit through the cervix (Tollner et al., 2011).

In human and rooster, the reduction of sperm Sia content is associated with lower fertility due to a migration default in the reproductive tract (Froman and Thursam, 1994; Tollner et al., 2011). Human seminal plasma and sperm acrosome contain other components that are supposed to counteract the female immune system such as *N*-glycans with SLe^x^ ending structure (Pang et al., 2007). Notably, polymers with a DP > 46 Sias residues directly and significantly reduce H1, H2A, H2B, H3, and H4 histones cytotoxicity (Zlatina et al., 2017). A glycomic gradient in the male genital tract fluids (from the cauda to the caput region of the epididymis), varying from high mannose to sialylated complex *N*-glycans is associated with abundant Le^x^ multimeric structures, and might also play an important role in the immune protection (Cheon and Kim, 2015). The human spermatozoa are suspended in the secreted seminal plasma composed of α2,6-sialylated branched *N*-glycans and Sias α2,3-linked *O*-glycans (Kratz et al., 2015). Spermatozoa are covered by sialylated fibronectin, detected to lower levels in infertile men (Kratz et al., 2011). The human epithelial cells of the epididymis caput are polySia-positive and polysialylated ST8SIA2 and polysialylated NCAM are integrated into the sperm membrane of the post-acrosomal region during the epididymal transit (Simon et al., 2013). Several ST3GAL4 (Taniguchi et al., 2003) or ST3GAL2 transcripts (Takashima et al., 2002; Krzewinski-Recchi et al., 2003) are detected in human testis, and postulated to be important for sperm Sias-coat acquisition. Sperm polySias may be cytoprotective from the innate and adaptive immune system of females. PolySias could bind Siglec 11 expressed on tissue macrophages, suppress the immune response (Wang and Neumann, 2010), and decrease the extracellular histones and nucleosomes cytotoxicity after their release from the neutrophils (Neutrophils extracellular traps) that have been detected after insemination (Alghamdi and Foster, 2005; Brinkmann and Zychlinsky, 2007). Intriguingly the X chromosomal type sperm exhibits a higher negative charge compared to the Y due to a difference in exposed Sias content (Kaneko et al., 1984).

Negatively charged Sias deliver signals through a binding to Siglecs (Li and Ding, 2019). Several Siglecs, present on the sperm surface displaying species- and regional-specific expression, are thought to be involved in sperm immune protection (Fernandez-Fuertes et al., 2018), such as Siglecs 1, 2, 5, 6, 10, and 14 expressed at the surface of, bull, ram, and human spermatozoa (Alkhodair et al., 2018), and Siglecs 11, and 16 present in human (Tecle et al., 2019). Bovine Siglecs 2, 5, 6, and 10 are expressed in the sperm neck region, and Siglecs 6 and 10, in the anterior head region (Alkhodair et al., 2018). Sperm has been found to strongly bind Siglecs present on immune cells which may facilitate sperm survival in the face of female immunity. In mice, the interaction of sialylated sperm to Siglec six predominantly present in B cells is positively correlated with protection from phagocytosis (Ma et al., 2012; Ma et al., 2016), and Siglec 3 in the endometria facilitates sperm survival (Tecle et al., 2019). In human, highly sialylated sperm binds Siglec 10 in the endometrium (Tecle et al., 2019), and a sperm Sia-containing glycoprotein, CD24 (a known activator of Siglec 10) (Chen et al., 2009)), interacts with endometrial expressed Siglec 10 and with neutrophil Siglec nine during the leukocytic reaction and increases sperm survival (Tecle et al., 2019). Glycodelin*-*A (GdA) secreted from the endometrial glands and the decidual glandular epithelium has two *N*-linked glycosylation sites and bears terminal α2,6-sialylated LacNAc and LacdiNAc antennae with immunosuppressive actions on cellular and humoral immune responses by blocking Siglec 2 (CD22) binding and activated-events mediated by T and B cells (Seppälä et al., 2007), (Figure 5). In addition to the Sias and Siglecs interaction between sperm and female genital tract, other mechanisms involving Sias may be involved in maternal immune regulation (Clark, 2013). In human, the high density of *N*-glycans terminated with a SLe^x^ are the ligands for Siglec nine and for selectins families of ligands, which is consistent with the hypothesis that human oocyte ZP glycans function in immune recognition (Clark, 2013). In addition, the female innate recognition is probably facilitated by the prevention of the complement deposition of factor H in the female reproductive tract (Brandtzaeg, 1997), as the binding of factor H to cell surface Sias could favor a conformational change that inhibits the alternative complement pathway activation and prevent the formation of the membrane attack and death complex.

### 4.2 Sperm Sias coat removal and mobility acquisition

#### 4.2.1 Sperm Sias and the storage in a female reservoir

In many animal species, sperm is stored by mated females before fertilization through several mechanisms. Some species have specialized storage organs or reservoirs, while others collect sperm in particular areas. Sperm storage within the female reproductive tract (up to months and years) is common in fish, bird, and amphibian females in oviductal sperm storage tubules or cloacal spermathecae (Sever et al., 2001). Tailed frog *Ascaphus truei* has oviductal sperm storage tubules while salamanders possess cloacal spermathecae (Sever et al., 2001). However, little is known about Sias function in this storage process in aquatic species. In birds, sperm transport and storage into tubules relies on Sias (Steele and Wishart 1996). In mammals, the oviduct is considered to be the sperm reservoir (Neubaum and Wolfner, 1999).

In mammals, sperm move to specialized regions that prolong viability and prepare for fertilization. Mammals oviductal sperm reservoir is an essential step to coordinate the sperm capacitation process and detachment from the oviduct in coordination with the appearance of the ovulated oocyte (Scott, 2000; Suarez, 2016). A few thousands from the millions of spermatozoa deposited in the female reproductive tract reach the oviduct where they bind in a Sia-dependent manner in the isthmic part to the ciliated ductal epithelial cells. The binding of spermatozoa involves Sias in hamster (Demott et al., 1995), rat (Cortés et al., 2004), and porcine where the sperm binds to specific oviductal α2,6-sialylated biantennary *N*-glycans (Kadirvel et al., 2012). In horse and bovine sperm-oviduct binding does not involve Sias but galactose (Sabeur and Ball, 2007), and fucose (Lefebvre et al., 1997) respectively. In human, the high sialylation of DEFB126 that coats the entire sperm surface is necessary for oviductal binding, extended viability, and capacitation retardation (Tollner et al., 2012). Sperm bound in a sialic acid-dependent manner to the isthmus of the oviductal epithelium is only detached to swim toward the oocyte in the ampulla of the oviduct when capacitation is induced (Suarez, 2016).

#### 4.2.2 Sperm capacitation unmasks Sias and triggers hyperactivation

Capacitation in aquatic species results from different types of interactions with either the surrounding external medium and the oocyte jelly, or can occur within the male genital tract. Sea urchin spermatozoa capacitation occurs in seawater due to a lower potassium concentration and a basic pH that enables the ATP hydrolysis for sperm motility (Tosti, 1994). In amphibians, the fibrous glycoconjugate network of the oocyte jelly layer containing Sias is involved in capacitation (Brun, 1974). In Fish, sperm capacitation is diverse and mediated either by seminal plasma changes in pH, HCO3, Na^+^, Ca^2+^, by release in the external medium, or after a contact with the oocyte surrounding Sias (Pérez, 2020).

In mammals, capacitation is also an important maturation process for spermatozoa (Aitken and Nixon, 2013). As ovulation occurs, spermatozoa undergoing capacitation shed their surface glycoproteins, reducing their binding to the oviduct epithelium, and start to develop hyperactivated motility. This increased mobility aids them to detach from the oviduct to further access the superior female reproductive tract (Töpfer-Petersen et al., 2002). In mouse, sperm releases sialidases that leads to the loss of 20% of the sperm sialome (Ma et al., 2012). In bull, the total removal of sialic acid from sperm decreases the motility and the mucus penetration ability but increases ZP binding and unwanted polyspermic penetration showing that the desialylation event requires fine-tuning (Fernandez-Fuertes et al., 2018).

In human, desialylation is restricted to a limited number of proteins, performed by sialidases present on the sperm surface (Ma et al., 2012), and likely facilitated by sialidases present in the female reproductive tract (Velasquez et al., 2007). Sperm neuraminidase 1 and 3 (Neu1 and Neu3) removing α2,3-, α2,6, and α2,8-linked Sias unmask certain sperm surface antigens required for the ZP recognition and binding (Lassalle and Testart, 1994; Ma et al., 2012). These sperm membrane-associated sialidases are activated by a specific Sia-binding protein found in the uterus (Banerjee and Chowdhury, 1997). Six proteins are desialylated (endothelial lipase, serine proteases 39 and 52, testis-expressed protein 101, and zonadhesin), while one becomes sialylated (aconitase), (Villaverde et al., 2020). The Sias of the *N*-glycan found on Asparagine 162 on aconitase is involved in a switch to glycolysis in capacitated sperm (Fraser and Lane, 1987). The remaining sialoproteins are relocated in the post-acrosomal region (Focarelli et al., 1995). In mouse as in human, capacitated spermatozoa gain increased motility called hyperactivation to leave the oviductal storage reservoir (Okabe, 2013). The CatSper1 component of the CatSper channel complex is an *O*-linked glycosylated protein that contains terminal Sias residues. The channel complex is compartmentalized within the flagellar membrane where it creates linear Ca^2+^ signaling nanodomains along the sperm tail to increase the mobility (Qi et al., 2007; Okabe 2013; Ded et al., 2020). Capacitation does not occur at the same time for all spermatozoa (Buffone et al., 2012). The oviduct regulates capacitation to avoid over-capacitation and premature acrosome reaction and ensure a continuous supply of fertile sperm (Boilard et al., 2002; Fazeli et al., 2003). It has been supposed that CatSper1 Sia-protein serves to select spermatozoa most likely to undergo capacitation (Ded et al., 2020). In human, a 25 kDa protein located on the non-capacitated sperm head plasma membrane binds to a Ca^2+^-dependent Sia-binding protein (SABP) present on the endometrium and contribute to increase sperm motility (Banerjee and Chowdhury, 1997), (Figure 5).

## 5 Fertilization and Sias

At fertilization, sperm binding results in acrosomal exocytosis, and sperm-ZP penetration, before gamete fusion (Mengerink and Vacquier, 2001). In several species, part of the Sias covering gametes as “biological masks” are removed to facilitate specific recognition of underlying glycans by specific binding proteins.

### 5.1 Sperm binding to the oocyte and sialoglycosylation

In spawning freshwater and marine organisms, the dilution that reduces the probability of gamete encounters is counteracted by the release of diffusible chemoattractant by the oocyte jelly (Bader, et al., 1998; Olson et al., 2001; Tholl et al., 2011; Guerrero et al., 2020). Once into close contact, the spermatozoa interact with the oocyte.

Sea urchin sperm (Miyata et al., 2004) interacts specifically with the oocyte jelly coat containing polySias and sulfated oligoSias chains inducing the acrosomal reaction (Yeşilyurt et al., 2015). These oligo/polySias act not only in sperm activation but are also involved in a species-dependent recognition by the release of a sperm major species-specific recognition effector called bindin after the contact with the oocyte sperm receptor and the subsequent acrosomal reaction (Lopo et al., 1982; Kitazume et al., 1996; Sato and Kitajima, 2013). The oocyte bindin receptors are aggregated into complexes in lipid rafts and the sperm polySias receptors mediate specific interactions (Hakomori, 2004; Cohen and Varki, 2010). However, the spermatozoa receptor for polyNeu5Gc is not identified and questions remain regarding carbohydrate-based sperm-oocyte recognition. In addition, polySias modify the spermatozoa intracellular pH and have been proposed to be involved in the extension of the acrosomal protrusion formed by actin filaments (Summers and Hylander 1975; Hirohashi et al., 2008).

In amphibians, except for some Urodeles where the sperm is released in the cloaca and stored in a spermatheca (Watanabe and Onitake 2002), fertilization occurs in the external medium. In newt Urodele, spermatozoa are directly inseminated on the oocyte jelly (rich in sialic acid and cations) before motility is triggered. In anuran, sperm motility is instead triggered by the external environmental composition changes and followed by a sperm-oocyte binding involving ZP, Sias and other glycoproteins.

Fish also show diverse reproductive strategies with most oviparous species having external fertilization, and few other zygoparous displaying internal fertilization. In several fishes having external fertilization, glycans can guide the spermatozoa to the oocyte into the micropylar canal playing a role similar to cell surface receptors. In the rainbow trout, *S. gairdneri*, oocytes express a 500 kDa Kdn-glycoprotein located in their vitelline envelope around the micropyle through which the spermatozoa can penetrate (Kanamori et al., 1990; Tezuka et al., 1994). This Kdn glycoprotein contains complex *N*-glycans as minor components and is heavily *O*-glycosylated with α2,8-linked Sias. In the cherry salmon vitelline envelope, the major component is a Neu5Gc mucin-type glycoprotein (Yanagimachi, 2011; Yanagimachi et al., 2017). Other teleost fishes present different sperm entry mechanisms through their micropyle being simply physical by an indented chorion around the micropyle in zebrafish and loach or requiring grooves in goldfish. In the case of flounder, herring, and Alaska pollock, the micropyle is a simple funnel devoid of sialoglycoprotein (Yanagimachi et al., 2017). The spermatozoa attached to the oocyte through the micropyle triggers the cortical alveoli discharge into the perivitelline space leading to PSGP cleavage, and an increase in the osmotic pressure which causes an influx of external water that swells the perivitelline space. This further ensures the chorion hardening and the closure of the micropyle to avoid polyspermy (Mengerink and Vacquier 2001; Gallo and Constantini, 2012). In the teleost Sebastidae blackbelly rosefish *Helicolenus dactylopterus,* the male possesses a urogenital copulating organ and fertilization occurs in the female genital tract where mucous cells from the papilla secrete sialoglycoproteins, *O*-linked mucin-type glycans terminated with α2,3-linked Sias, and mannose type *N*-linked glycans terminated with sialic acid α2,6-linked Sias to galactose/*N*-acetylgalactosamine, which role remains to be clarified (Accogli et al., 2018).

In mammals, fertilization takes place in the ampulla of the fallopian tubes, and sperm binding to the oocyte occurs in the ZP made of *N*- and *O*-glycoproteins (Tecle and Gagneux, 2015). The molecular model proposed to explain sperm-oocyte interaction in mammals has changed through the years, going from ZP2 and ZP3 acting as sperm receptors (Bleil and Wassarman, 1980; Bleil and Wassarman, 1988; Bleil and Wassarman, 1990), to the possibility that sperm interaction with the ZP depends on the supramolecular structure of the oocyte coat rather than a single ZP subunit (Rankin et al., 2003), to a more recent view with one domain of ZP2 playing a central role in gamete recognition by physically interacting with counterpart molecule(s) on sperm (Avella et al., 2014). Currently available data do not fully exclude the role of ZP glycosylation and Sias in fertilization in one of its multistep processes such as the formation of the membrane block to polyspermy, or ZP hardening, but this involvement differs from what was initially suggested and varies between species.

In pig, gamete recognition depends more on *N*- than *O*-glycans (Noguchi et al., 1992; Yonezawa et al., 1997), and particularly the main ZP-neutral di-antennary *N*-glycans (Nakano et al., 1996; Topfer-Petersen, 1999). The *N*-glycosylation of ZP glycoproteins was shown necessary for sperm-ZP interaction, including sperm binding to ZP as discussed in chapter 3.2 (Lay et al., 2013).

In bovine, Sias of the ZP are physiologically implicated in the binding of the sperm to the ZP (Velasquez et al., 2007).

Mice genetically modified, lacking *N*- and *O*-glycans (and without Sias), are fertile as discussed in chapter 3.2, showing sperm binding is independent of *N*- and *O*-glycans from the ZP (Thall et al., 1995; Shi et al., 2004; Williams et al., 2007; Raj et al., 2017; Tokuhiro and Dean, 2018). *N*-glycosylation was shown important for the normal secretion of ZP2 (Roller and Wassarman, 1983).

For human, terminal SLe^x^ sequences, present on human ZP, are not essential for sperm binding as discussed in chapter 3.2 (Miranda et al., 1997; Oehninger et al., 1998; Pang et al., 2011; Bhakta et al., 2019; Tumova et al., 2021), and requires other molecular effectors (Siu et al., 2021), even if lectin-like and protein–protein interactions were initially shown to play a role in gamete interaction (Clark, 2013). Human ZP2 without SLe^x^ antigen, expressed in transgenic mice, is sufficient for human sperm binding and penetration in the ZP (Baibakov et al., 2012; Avella et al., 2014). Chimeric ZP2 proteins composed of the human N-terminus instead of the mouse terminal region allow human sperm binding showing a role for this region in species-specificity (Avella et al., 2014; Tokuhiro and Dean, 2018).

Interestingly, in Drosophila, oocytes are surrounded by a thick protective chorionic eggshell that presents a cone-shaped specialized projection, or micropyle, through which the sperm enters at fertilization as in teleosts (Horne-Badovinac, 2020). While *Drosophila* polySias homopolymers are present during embryonic development, no Sias nor polySias glycoproteins were detected before fertilization in oocytes and spermatozoa (Koles et al., 2004). It is hypothesized that Sias could be unnecessary at fertilization or act in a pre-mating step with a preferential selection of the spermatozoa through transport and storage in the specialized female spermatheca. This selection would build an efficient reproductive barrier by preventing heterospecific fertilization by a sperm selection before gamete encounter (Intra et al., 2009).

### 5.2 Sperm acrosomal reaction, oocyte deprotection, and membrane block to polyspermy

In sperm, the outer acrosomal membrane at the apical tip of the head fuse with the plasma membrane allowing exocytosis of the acrosomal contents originating from the spermatid’s Golgi complex and containing a variety of lytic enzymes and ZP-binding proteins including acrosin, hyaluronidase, and sialidases (Srivastava et al., 1988; Yanagimachi, 2011; Khosravi et al., 2016). Acrosome-reacted sperm digest the protective oocyte vestment and bind to the oocyte plasma membrane (Okabe, 2015). Upon contact with the spermatozoa, several vertebrates and invertebrates oocytes exhibit polyspermy-preventing reactions. The membrane block to polyspermy is a rapid reaction that converts the oocyte jelly coat to a form that does not support sperm interaction. Urodeles amphibians, cartilaginous fish, and birds accept polyspermy. The total content of Sias between the vitelline membrane compared to the fertilization membrane does not vary (Wolf et al., 1976). It is not known if this absence of level changes is restricted to some species and if it reflects the sum of sialylation/desialylation processes or is simply a low level of Sias dynamical change.

In sea urchin, as seen in chapter 3.1.2, the acrosomal reaction is potentiated by fucose-sulfated polymers and also requires the oocyte Sia-rich polysaccharides found on the 350 kDa sperm receptor that belongs to the heat shock protein 110 family (Kitazume et al., 1996; Hirohashi et al., 2008; Sato and Kitajima, 2013; Yeşilyurt et al., 2015). The oocyte 350 kDa sperm-binding protein is largely localized in the vitelline layer and lipid rafts and acts for a large part in the acrosome reaction (Maehashi et al., 2003). In sea urchin and non-mammalian species, the membrane block to polyspermy is mediated by the exocytosis of oocyte cortical granules to convert and elevates the oocyte jelly coat. It is accompanied by multiple biochemical changes and by a transient depolarization of the oocyte plasma membrane potential (Jaffe and Cross 1986; Longo et al., 1986; McCulloh and Chambers, 1992).

In mammals, sperm capacitation destabilizes the acrosomal sperm head membrane for penetration in the outer layer of the oocyte in addition to the chemical changes in the tail necessary for greater mobility. Sialylated acrosin released from the sperm acrosome digests the ZP allowing part of the sperm plasma membrane to fuse with the egg plasma membrane, in the boar (Schleuning et al., 1975), or hamsters (Hirose et al., 2020). Acrosin homologs have been identified in quail bird (Sasanami et al., 2011), and *Xenopus* (Kubo et al., 2010) to bind to the oocyte. However, in mice, acrosin null mutation did not impair fertilization indicating it is not a necessary molecular effector in this process (Wassarman, 2011). The polyspermy-preventing reaction in mammals is composed of complex slow calcium-dependent reactions in the oocyte jelly and plasma membrane, requiring cortical granule exocytosis and ZP conversion (Evans, 2020). The cortical granules exocytosis allows ZP mechanical properties to change from elastic to plastic, also called hardening (Papi et al., 2010; Gupta and Bhandari, 2011; Fahrenkamp et al., 2020). However, to prevent polyspermy mammals’ oocytes have been observed to preferentially use a ZP coat block or a plasma membrane block. In sheep or dog, no sperm is found in the perivitelline space, between the ZP and the plasma membrane, suggesting an effective ZP block while in some other species such as rabbit, or mole sperm is found in the perivitelline space suggesting a plasma membrane block rather than a ZP block (Evans, 2020).

In pig, the N-glycosylation of ZP glycoproteins was shown necessary for the induction of acrosomal exocytosis in the ZP-bound sperm (Lay et al., 2013). The number of oocyte Sias decreases after fertilization (Rath et al., 2005), and after the exocytosis of cortical granules (Sun, 2003). Desialylation of the oocyte decreases sperm acrosome reaction and polyspermy block (Lay et al., 2011).

In bovine, it was hypothesized that neuraminidase released from the cortical granules would participate in the polyspermy block by removing Sias from the ZP (Velasquez et al., 2007). Sias need to be removed from bovine oocyte coat to allow sperm interaction but are required to avoid abnormal fertilization (Fernandez-Fuertes et al., 2018), pointing to a fine-tuned regulation of Sias.

In mice, two sperm sialidases (neuraminidases 1 and 3), shed during capacitation, interfere with ZP binding but are not associated with the polyspermy block (Ma et al., 2012). By using a transgenic mouse line expressing GFP in the acrosome, it was determined that most sperm undergo an acrosome reaction before contacting the ZP *in vitro* (Hirohashi et al., 2011; Jin et al., 2011). The timing of the acrosome reaction before the ZP binding is flexible, and exocytosed enzymes are dispensable for sperm penetration in the ZP (Okabe, 2015). The majority of sperm has undergone acrosome exocytosis in the upper segments of the oviductal isthmus before the ampulla (La Spina et al., 2016) and the cumulus (Hino et al., 2016; Muro et al., 2016), suggesting that sperm acrosome exocytosis could take place in the female oviduct during sperm migration, and questioning about the role played by Sias in the female reproductive tract.

In human, terminal SLe^x^ sequences have been involved in the sperm acrosomal reaction (Miranda et al., 1997; Oehninger et al., 1998; Pang et al., 2011; Tumova et al., 2021). Recently a protein bearing terminal SLe^x^, C1orf56, localized in the human acrosomal region of the spermatozoa was proposed to act in the ZP-induced acrosome reaction (Wang et al., 2021). In addition, three of the four reported glycodelin (Gd) sialylated glycoproteins (acquired by the sperm in the female genital tract) GdA, GdF, and GdC, but not GdS coating the spermatozoa, are involved in the regulation of the acrosomal reaction. GdF blocks premature acrosomal reaction during Fallopian tube migration. GdF is subsequently converted into GdC by the oocyte cumulus cells when a sperm-oocyte contact is established, removing GdF inhibition (Sawyer, 2021).

## 6 Conclusion

In reproduction, there is a need to allow fertilization between different individuals in a given species, with the prevention of unwanted cross-fertilization by other related species, and a limited microbial attack. Cell-surface sialoglycoproteins coating germ cells show sufficient diversity to fulfill these recognition/restriction aspects even if they are not the sole type of post-translational modifications involved in this process. Sialylated glycoproteins display a large repertoire and have a spatial organization to allow several types of complex interactions with clustering in species with internal fertilization and polySias chains in species with external fertilization. Sialoglycoproteins are involved in many steps before fertilization including sperm maturation and are part of the molecular basis of gamete binding in several species. In mammals and fish with internal fertilization, sialylated glycoproteins have an emerging role in the passage and attachment of sperm through the female reproductive tract where they fulfill a protective action against female immunity that starts to be understood. The identification of specific molecules that mediate sperm storage would help clarify how sperm can be kept viable. In free spawning organisms submitted to a dilution in the external media, polySias chains play important roles in terms of pathogens/pollutants and as self/non-self-recognition mechanisms.

However, many important issues related to Sias terminal residues in the surrounding gametes matrix remain unresolved. For example, what is the role of ZP3 Sias in mammals other than mice, and do they act in immune recognition? Why are polySias present in aquatic species and what is their relevance compared to other shorter forms of terminal sialic acids in terrestrial species? Are Sias mainly involved in immune recognition, or do they act in membrane fusion by favoring membrane attachment to different proteins located in multimeric complexes? Defects in the formation of microdomains at the level of the human oolemma plasma membrane have been demonstrated to be related to fertilization failure (Van Blerkom and Caltrider, 2013).

To generate a sufficient level of complexity, sialylated glycoproteins present at the cell surface are synthesized in the Golgi apparatus in a process subjected to multiple sequential and competitive enzymatic pathways (Drickamer and Taylor, 1998; Esko and Selleck, 2002). The expression of sialylated glycoproteins is regulated by a limited set of conserved sialyltransferases in terms of orthologs and enzymatic activity (Harduin-Lepers et al., 2005) providing the basis for a rationale for their expression (Varki, 2006; Yamakawa et al., 2018).

More than ever, the determination of gene expression patterns and activity profiles of the enzymes involved in sialoglycoproteins biosynthesis is a requisite to better understand their biological functions and interconnections in gametes and at fertilization. In this respect, further studies on sialyltransferases in several species using newly developed technology, such as CRISPR-Cas9, linked to an analysis of the gonads could provide future valuable information.

## Abbreviations

CAZy, Carbohydrate Active EnZymes database; CMAH, N-acetylneuraminic acid hydroxylase; CMAS or CSS: CMP-sialic acid synthetase; CMP, cytidine monophosphate; GalNAc, N-acetylgalactosamine; GlcNAc, N-acetylglucosamine; Gd, glycodelin; GH33, glycoside hydrolase 33; GNE, glucosamine UDP-GlcNAc-2-epimerase/N-acetylmannosamine kinase; Neu, neuraminic acid; GT29, glycosyltransferase family 29; Kdn, 2-keto-3-deoxy-d-glycero-d-galacto-nonulosonic acid; NANP, N-acetylneuraminate-9-phosphatase; NANS, Neu5Ac-9-P synthase; NEU, neuraminidase; Neu5Ac, N-acetylneuraminic acid; Neu5Gc, N-glycolylneuraminic acid; NCAM, neural cell adhesion molecule; polySias, polysialic acids; PB, polar body; PEP, phosphoenolpyruvate; PSGP, polysialoglycoprotein; Sd^a^. antigen, (Neu5Acα2,3(GalNAc β1,4)Galβ1,4GlcNAc); Sia, sialic acid; SLe^x^, Sialyl-Lewis(x) (NeuA5cα2,3Galβ1,4(Fucα1,3)GlcNAc); ST8SIA2, Alpha-N-Acetyl-Neuraminide Alpha-2,8-Sialyltransferase two; ST8SIA4, Alpha-N-Acetyl-Neuraminide Alpha-2,8-Sialyltransferase four; UDP, uridine diphosphate; WGDR, round of whole genome duplication; ZP, zona pellucida.
